# Celiac Anti-Type 2 Transglutaminase Antibodies Induce Phosphoproteome Modification in Intestinal Epithelial Caco-2 Cells

**DOI:** 10.1371/journal.pone.0084403

**Published:** 2013-12-31

**Authors:** Gaetana Paolella, Ivana Caputo, Anna Marabotti, Marilena Lepretti, Anna Maria Salzano, Andrea Scaloni, Monica Vitale, Nicola Zambrano, Daniele Sblattero, Carla Esposito

**Affiliations:** 1 Department of Chemistry and Biology, University of Salerno, Salerno, Italy; 2 European Laboratory for the Investigation of Food-Induced Diseases (ELFID), University “Federico II”, Naples, Italy; 3 Proteomics and Mass Spectrometry Laboratory, ISPAAM, National Research Council, Naples, Italy; 4 Department of Molecular Medicine and Medical Biotechnologies, University “Federico II”, Naples, Italy; 5 CEINGE Biotecnologie Avanzate, Naples, Italy; 6 Department of Health Sciences and IRCAD, University of Eastern Piedmont, Novara, Italy; Tulane University, United States of America

## Abstract

**Background:**

Celiac disease is an inflammatory condition of the small intestine that affects genetically predisposed individuals after dietary wheat gliadin ingestion. Type 2-transglutaminase (TG2) activity seems to be responsible for a strong autoimmune response in celiac disease, TG2 being the main autoantigen. Several studies support the concept that celiac anti-TG2 antibodies may contribute to disease pathogenesis. Our recent findings on the ability of anti-TG2 antibodies to induce a rapid intracellular mobilization of calcium ions, as well as extracellular signal-regulated kinase phosphorylation, suggest that they potentially act as signaling molecules. In line with this concept, we have investigated whether anti-TG2 antibodies can induce phosphoproteome modification in an intestinal epithelial cell line.

**Methods and Principal Findings:**

We studied phosphoproteome modification in Caco-2 cells treated with recombinant celiac anti-TG2 antibodies. We performed a two-dimensional electrophoresis followed by specific staining of phosphoproteins and mass spectrometry analysis of differentially phosphorylated proteins. Of 14 identified proteins (excluding two uncharacterized proteins), three were hypophosphorylated and nine were hyperphosphorylated. Bioinformatics analyses confirmed the presence of phosphorylation sites in all the identified proteins and highlighted their involvement in several fundamental biological processes, such as cell cycle progression, cell stress response, cytoskeletal organization and apoptosis.

**Conclusions:**

Identification of differentially phosphorylated proteins downstream of TG2-antibody stimulation suggests that in Caco-2 cells these antibodies perturb cell homeostasis by behaving as signaling molecules. We hypothesize that anti-TG2 autoantibodies may destabilize the integrity of the intestinal mucosa in celiac individuals, thus contributing to celiac disease establishment and progression. Since several proteins here identified in this study were already known as TG2 substrates, we can also suppose that transamidating activity and differential phosphorylation of the same targets may represent a novel regulatory mechanism whose relevance in celiac disease pathogenesis is still unexplored.

## Introduction

Type 2 transglutaminase (TG2), also named tissue TG, is a multifunctional enzyme widely distributed in mammalian cells and tissues. TG2 biological functions are strictly correlated to its intracellular or extracellular localization [1]. The main characterized enzymatic activity consists of the calcium-dependent isopeptide-bond formation between the γ-carboxamidic group of glutamine and the ε-amino group of lysine; both amino acids can belong to several intra- or extracellular proteins [2], [3]. In the absence of available amines, TG2 can deamidate specific endoproteic glutamines to glutamic acid [4]. By means of its transamidating activity, TG2 contributes to stabilizing the extracellular matrix and modulates cell-matrix interactions and cell adhesion [5]. Within cells, TG2 is strictly regulated by calcium availability and it can crosslink many substrates during necrosis and during the final stages of apoptosis [2]. TG2 can also bind and hydrolyze GTP, thus acting in signalling pathways associated with phospholipase δ-C [6]. Furthermore, both kinase and disulphide-isomerase activities have been attributed to TG2 [7], [8]; however, the function of such activities is poorly understood. Finally, the membrane-bound TG2 acts as a co-receptor for fibronectin together with the extracellular domain of β1/β3 integrins, in a catalytic-independent manner [9] and also forms complexes with some membrane tyrosine kinase receptors [10].

Growing experimental evidences indicate that TG2 is involved in several human pathologic conditions such as cancer, neurodegenerative diseases, fibrosis and autoimmune diseases [11], [12]. In particular, TG2-catalyzed deamidation of specific glutamines in dietary gliadin, the main proteic component of wheat, seems to play a key role in the pathogenetic mechanism of celiac disease (CD), an inflammatory condition of the small intestine characterized by a specific immune response to peptides derived from ingested gliadin in genetically-susceptible individuals [13], [14]. Some studies have reported an increased expression of TG2 in CD mucosa, with respect to normal subjects, particularly at the level of the *lamina propria* and in enterocytes [15]–[17]. However, mucosal TG2 level cannot be considered a diagnostic marker for CD, as biopsies from patients with chronic duodenitis or Chron's disease display a similar immunohistochemical pattern for TG2 [15], [18]. In line with these findings, it has been demonstrated that exposure to inflammatory stimuli may induce TG2 upregulation [19]. In addition, TG2 overexpression and activation is also induced by toxic gliadin peptides [20], [21]. It has been proposed that increased TG2-transamidating activity in the CD mucosa could also be responsible for the strong autoimmune response in CD, TG2 being the main autoantigen, through the formation of crosslinks between gliadin and TG2 itself and the consequent stimulation of TG2-specific, normally silent, B lymphocytes [22]. Anti-TG2 IgA deposits in the CD mucosa appear in the very early phase of the disease and they can successively spill over into the blood from the intestine when the mucosa is still intact [23]. Interestingly, even seronegative patients have mucosal anti-TG2 deposits when on a gliadin-containing diet [24]. Anti-TG2 antibodies disappear during a gliadin-free diet but rapidly reappear when gliadin is reintroduced into the diet of CD patients. For this reason, circulating anti-TG2 antibodies represent an important serological marker of active CD.

We previously demonstrated that anti-TG2 autoantibodies can reduce TG2 catalytic activity [25]. As a consequence, antibodies can interfere with TG2-mediated repair of the damaged mucosa [26]. In addition, some studies have highlighted the ability of anti-TG2 antibodies to direct out-in signalling in different cell models through the interaction with the membrane-bound TG2 [27], [28]. We also reported that celiac anti-TG2 antibodies were able to induce ERK phosphorylation and calcium mobilization from intracellular stores in intestinal epithelial cells [29], [30].

In the present study, we aimed to understand better the signalling pathways that are triggered by anti-TG2 antibodies. We have studied the phosphoproteome modifications in intestinal epithelial cells stimulated by celiac anti-TG2 autoantibodies. We have used a two-dimensional electrophoresis (2-DE) approach to separate total proteins and, after specific staining of phosphoproteins, mass spectrometry (MS) analysis identified several components showing changes in phosphorylation level. Interestingly, we demonstrate a differential phosphorylation of proteins mainly belonging to the class of chaperones and of cytoskeletal proteins, or involved in signalling and metabolic processes. The analyses of the intracellular response induced by anti-TG2 antibodies may contribute to gain a deeper knowledge of possible pathogenetic role of anti-TG2 antibodies in CD.

## Methods

### Cell Culture and Antibodies Treatment

Human colorectal adenocarcinoma cells (Caco-2) were obtained from Interlab Cell Line Collection (Centro di Biotecnologie Avanzate, Genova, Italy). Caco-2 cells were cultured in Dulbecco's modified Eagle's medium supplemented with 10% fetal bovine serum, 1% non-essential amino acids, 0.2 mM L-glutamine, 50 units/ml penicillin and 50 µg/ml streptomycin (Invitrogen SRL, Milan, Italy). Cells were maintained at 37°C in a 5% CO_2_, 95% air-humidified atmosphere.

Antibody treatments were performed as previously described [29]. Briefly, cells were plated at 1.5×10^4^ cell/cm^2^ and 24 h later were cultured for 72 h in medium containing 0.1% fetal bovine serum. The cells were then incubated for 5 min with the recombinant anti-TG2 miniantibody clone 2.8 (2 µg/ml) or with affinity purified mouse IgG (2 µg/ml) (Santa Cruz Biotechnology Inc., CA, USA). Clone 2.8 was derived from a library of CD intestinal lymphocytes. The cloning procedure has been reported elsewhere [31]. Briefly, a 2.8 single chain antibody was subcloned from the pDAN vector to a scFv-Fc expressing vector engineered to contain the Hinge-CH2-CH3 domains of mouse IgG1. CHO cells were transfected to produce stable clones secreting 2.8 scFv-Fc. Miniantibodies produced in the medium of an isolated cell clone were affinity purified by using an HiTrap protein G column (GE Healthcare, Milan, Italy). Table S1 summarizes previously reported data on biological effects and properties of clone 2.8 with respect to mouse IgG here employed, and with respect to the well characterized commercial mouse anti-TG2 antibody CUB 7402.

### Two-dimensional Electrophoresis (2-DE)

To obtain cells extracts, Caco-2 cells were washed twice with phosphate-buffered saline (PBS) and lysed in 50 mM Tris pH 7.4 containing 1 mM EDTA, 5 mM MgCl_2_, 150 mM NaCl, 1% Triton X-100, 1 mM Na_3_VO_4_, 1 mM NaF, 1 mM phenylmethylsulfonyl fluoride and proteases inhibitors (Sigma-Aldrich, Milan, Italy). Lysates were clarified by centrifugation at 12,000×*g* for 30 min, at 4°C.

For 2-DE, Caco-2 cells extracts were precipitated overnight in 10 vol of ice-cold precipitation solution (10% v/v methanol in acetone) at −20°C overnight, and recovered by centrifugation at 16,000×*g* for 30 min, at 4°C. 150 µg of precipitated proteins were dissolved in rehydration buffer containing 7 M urea, 2 M thiourea, 4% CHAPS, 0.1% ampholyte Bio-lyte 3/10 (Bio-Rad Laboratories, Milan, Italy), 30 mM dithiothreitol and 0.002% bromophenol blue (Sigma-Aldrich). Solubilised proteins in rehydration buffer were used to actively hydrate immobilized pH gradient IPG gel strips (7 cm, pH 4.0–7.0, pH 3.9–5.1, pH 4.7–5.9, pH 5.5–6.7) for 12 h, at 20°C with current of 50 µA/IPG strip using the Protean IEF Cell (Bio-Rad Laboratories). Running conditions were set at 250 V for 15 min, 4000 V for 2 h, 500 V for 2 h with maximum current of 50 µA/IPG strip. After isoelectrofocusing, strips were sequentially equilibrated in equilibration buffer (6 M urea, 2% sodium dodecyl sulphate, 20% glycerol, 0.375 M Tris-HCl, pH 8.8) for 10 min in the presence of 0.5% dithiothreitol, and for 10 min in the presence of 2.5% iodoacetamide. 2-DE were run on 10% sodium dodecyl sulphate polyacrylamide gel for 1 h at constant 120 V. Finally, 2-D gels were subjected to fluorescent staining or to western blot analysis.

### Fluorescent Gel Staining

Gels were fixed into 50% methanol/10% acetic acid, overnight, washed with distilled water and then incubated with the phosphoproteins-specific reagent Pro-Q Diamond (Invitrogen) for 90 min, in the dark. Gels were destained in a buffer consisting of 50 mM sodium acetate, pH 4.0 and 20% acetonitrile for 30 min. To detect total proteins, gels were stained with the SyproRuby fluorescent dye (Invitrogen) overnight, in the dark, and then washed with 10% methanol/10% acetic acid.

### Image Analysis

Gel images of phosphoproteins were obtained by scanning 2-D gels with a Typhoon 9400 laser scanner (GE Healthcare) with excitation at 532 nm and emission at 560 nm. A gel image of total proteins was obtained by scanning with excitation at 450 nm and emission at 610 nm. Gels images were analyzed with the software PDQuest version 7.3.1 (Bio-Rad Laboratories). A match-set was created from the protein patterns of triplicate 2-D maps. A standard gel was generated out of the image with the best average spots quality. Spots were detected by creating a three-dimensional Gaussian spot; all gels were normalized to remove variations that were not due to differential phosphorylation of proteins in the spot. Raw intensity of Pro-Q Diamond-stained spots in a gel was divided by the intensity of SyproRuby-stained corresponding spots. The ratio between normalized obtained values relative to anti-TG2 treated samples and normalized obtained values relative to IgG-treated samples, respectively, was reported as fold change.

### Protein Identification

Spots from 2-DE were picked by a robotic picker (Ettan spot picker, GE Healthcare), then triturated and washed with water. Proteins were *in-gel* reduced, S-alkylated, and digested with trypsin, as previously reported [32]. Protein digests were subjected to a desalting/concentration step on microZipTipC18 pipette tips (Millipore Corp., Bedford, MA, USA) before nano-LC-ESI-LIT-MS/MS analysis. Samples were analyzed with a LTQ XL mass spectrometer (Thermo, San Jose, CA, USA) equipped with a Proxeon nanospray source connected to an Easy-nanoLC (Proxeon, Odense, Denmark). Peptide mixtures were separated on an Easy C18 column (100×0.075 mm, 3 µm particle size) (Thermo) by using a linear gradient from 5% to 50% of acetonitrile in 0.1% formic acid, over 60 min, at a flow rate of 300 nl/min. Spectra were acquired in the range *m/z* 400–2000. Acquisition was controlled by a data-dependent product ion scanning procedure over the three most abundant ions, enabling dynamic exclusion (repeat count 2 and exclusion duration 1 min). The mass isolation window and collision energy were set to *m/z* 3 and 35%, respectively. MASCOT software package version 2.3.02 (Matrix Science, UK) was used to identify spots from a database of human protein sequences. Nano-LC-ESI-LIT-MS/MS data were searched by using a mass tolerance value of 2.0 Da for a precursor ion and 0.8 Da for MS/MS fragments, with trypsin as proteolytic enzyme, a missed-cleavage maximum value of 2, Cys carbamidomethylation and Met oxidation as fixed and variable modifications, respectively. Protein candidates with more than 2 assigned peptides with an individual MASCOT score >30 were further evaluated by comparison with their calculated mass and pI values, using the experimental values obtained from 2-DE (see Table S2 for details).

### Western Blotting

Protein lysates prepared from *ex-novo* independent experiments were used to perform western blotting validation. After 2-DE, proteins were electro-transferred to PVDF membranes (Millipore Corp.). Then, nonspecific binding sites were blocked in PBS containing 5% non-fat dry milk, and membranes were incubated overnight with specific mouse monoclonal antibodies recognising elongation factor 1-γ (EF1γ) (clone X5-P), translationally-controlled tumor protein (TCTP) (clone B3), 60 kDa heat shock protein (HSP60) (clone 24) (Santa Cruz Biotechnology Inc.), at a dilution of 1∶1000 in PBS containing 1% non-fat dry milk. Membranes were washed with PBS containing 0.1% Tween-20 and incubated with anti-mouse horseradish peroxidase-conjugated secondary antibodies for 1 h, at room temperature. Immunocomplexes were revealed using a chemiluminescence detection kit (EuroClone, Milan, Italy), according to the manufacturer’s instructions. To compare specific signals from different blots, we stained all membranes with the Colloidal Gold Total Protein stain (Bio-Rad Laboratories), then aligned at least three different unspecific spots with the same position in each blot.

### Bioinformatics Analysis

The information about known phosphorylation sites was retrieved from UniProt database [33], selecting only the information for which a direct reference for human proteins was available (information inferred by similarity was discarded), and from PhosphoSite Plus Web resource [34], selecting the information for human proteins supported by at least one publication (when more than 5 references were present, the phosphorylation site was considered to be highly reliable). In addition, predictions of phosphorylation sites was made using NetPhos2.0 Web Server [35], selecting the phosphorylation sites predicted with a threshold higher than 0.7 (those predicted sites with a threshold higher than 0.9 were considered highly reliable) and PHOSIDA server [36] using the predictor specific for human proteins, with default precision of 95%. Moreover, NetPhosK 1.0 Web Server [37] was used to predict kinase-specific phosphorylation, by applying the method with Evolutionary Stable Sites (ESS) Filter, and again selecting the phosphorylation sites predicted with a threshold higher than 0.7, and filtering out phosphorylation sites not positively predicted in a greater subset of the homologues.

For those proteins validated by western blot, an evaluation of the position of the putative phosphorylation sites was made based upon their structures available in PDB database [38] (for the C-terminal domain of EF1γ and TCTP), or on the models obtained by automatic homology modelling procedures and available in Protein Model Portal [39] (for the N-terminal domain of EF1γ and HSP60). A manual inspection of the structures was made using PyMOL (http://www.pymol.org).

The evaluation of the functional categories of identified proteins was made using PANTHER [40], and, when data were not available, they were retrieved from UniProt. The analysis of the biological pathways in which each protein is involved was made using KEGG [41]. The analysis of protein-protein interactions was made using PSICQUIC View Web server (http://www.ebi.ac.uk/Tools/webservices/psicquic/view/main.xhtml).

## Results

### Identification of Differential Phosphoproteins in Anti-TG2-stimulated Cells and Control Cells

**Figure 1:**
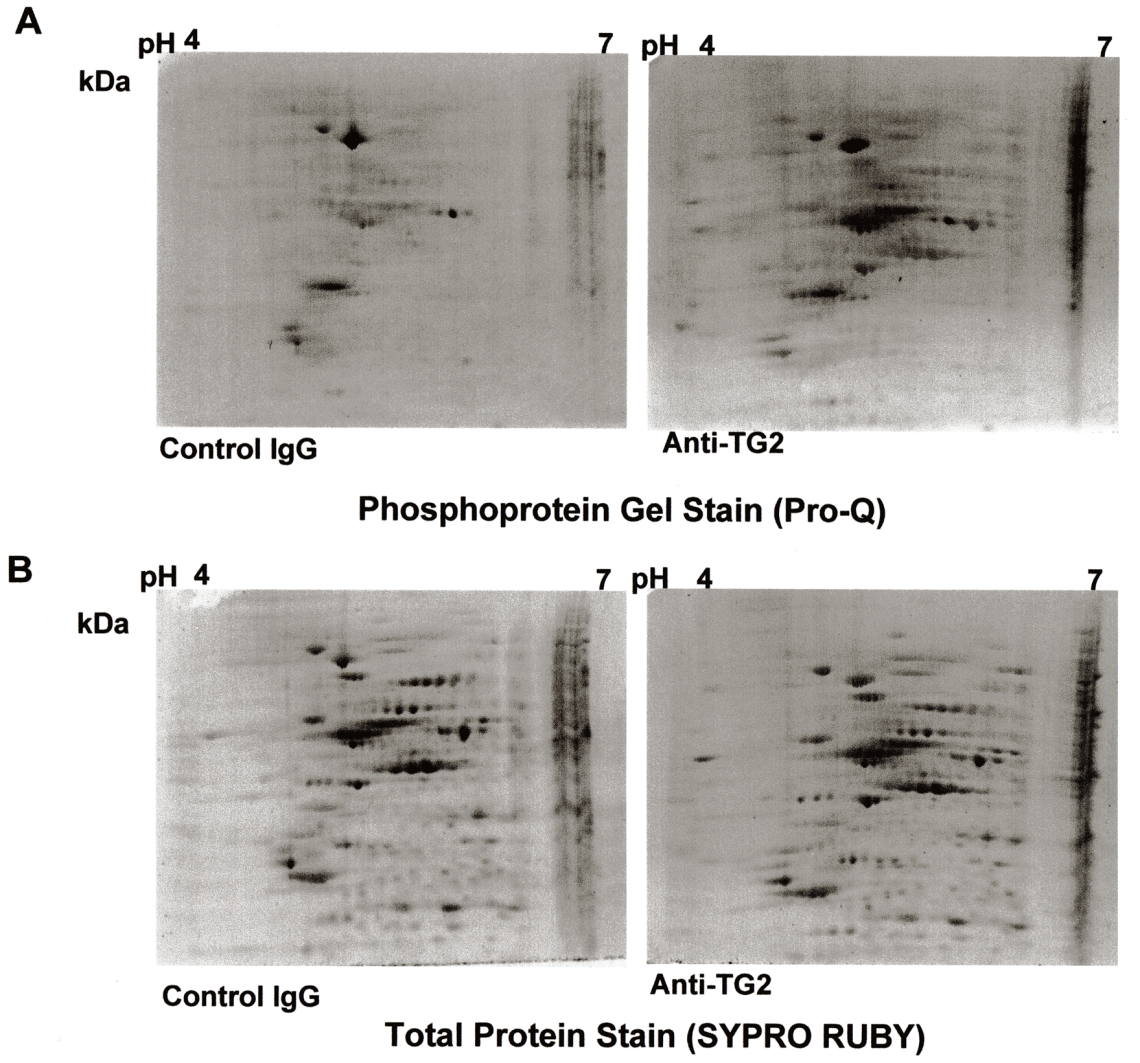
Representative 2-D-maps of phosphorylated and total proteins from Caco-2 cells treated with anti-TG2/control antibodies. Total protein extracts (150 µg) were separated on linear IPG strips 7 cm, pH 4.0–7.0, followed by 10%-sodium dodecyl sulphate polyacrylamide gel electrophoresis. 2-D gels were stained with the phosphoprotein-specific reagent Pro-Q Diamond (**A**) and with the SyproRuby dye to detect total proteins (**B**). 2-D-maps are representative of three biological independent replicates.

Since we demonstrated that a brief treatment with anti-TG2 antibodies induced ERK-phosphorylation in Caco-2 cells [29], we performed a 2-D-phosphoproteome analysis on protein cellular extracts to identify other proteins differentially phosphorylated. Analyses were performed on three biological replicates; representative 2-D profiles are shown in Figure 1. Gels were first stained with the Pro-Q Diamonds phosphoprotein stain (Figure 1A) and then with the SyproRuby total protein stain (Figure 1B). The sequential staining procedure enabled the calculation of the relative phosphorylation level of each corresponding spot and thus the detection of differential phosphorylation.

**Figure 2:**
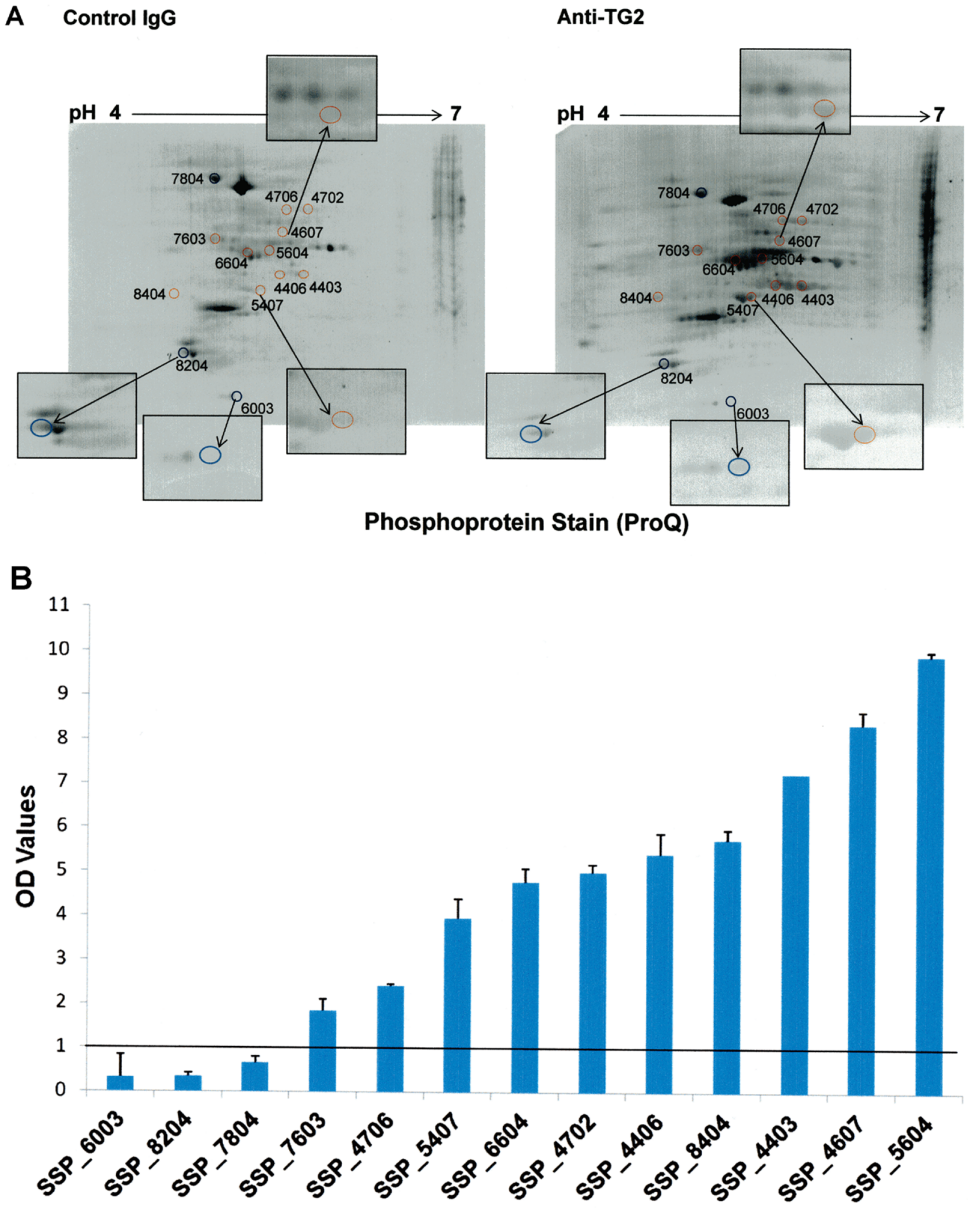
Selected spots for MS analysis. (**A**) Coloured circles indicate the position on Pro-Q Diamond-stained gels of the 13 protein spots picked for MS analysis. Red circles are associated to hyperphosphorylated proteins; blue circles are associated to hypophosphorylated proteins. Boxes show an enlargement of picked spots. (**B**) Fold change values for the 13 selected spots. All the spots were differentially stained in anti-TG2-treated cells with respect to control IgG-treated cells. *P<0.05* (Student’s t-test).

Approximately 400 protein spots were resolved in all gels stained with SyproRuby. Analysis performed by PDQuest on 2-D maps identified 114 phosphorylated spots (28% of total spots); 53 of which were differentially stained in anti-TG2-treated cells with respect to unspecific antibody-treated cells (*P value* <0.05, Student’s t-test). In particular, 43 spots showed a higher phosphorylation level and 10 spots showed a lower phosphorylation level in anti-TG2-treated cells. Among these, we selected those spots (13 in number) showing highest changes in phosphorylation status for further MS analyses (Figure 2A). In particular, 3 of them showed fold change values lower than 0.7 in TG2-treated samples with respect to the control samples, whereas the other 10 showed fold changes values higher than 1.8 with respect to the control samples (Figure 2B). We identified 14 proteins that are reported in Table 1. Among these, TCTP, endoplasmin and 14-3-3 protein ε, present in 3 different spots, appeared hypophosphorylated in response to TG2-antibodies treatment. Nine proteins, namely beta actin variant, mitochondrial HSP60, HSP70-8 isoform 2 variant, creatine kinase M-type, tubulin beta-2A chain, protein disulfide-isomerase (PDI), EF1γ and two uncharacterized proteins, present in 9 different spots, appeared hyperphosphorylated in response to TG2-antibodies treatment. Finally, two proteins, i.e. beta 5-tubulin and tubulin alpha-1A chain, were found into the same spot, which appeared to have a higher phosphorylation level in TG2-treated samples with respect to the control samples.

**Table 1:** MS analysis and database searching of the 13 spots with differential phosphorylation level in samples from anti-TG2 treated cells and from control-IgG treated cells.

Spot number	UniProtProtein ID	UniProt ProteinAccession Code	Protein name	MASCOT Score	ProteinMass	Protein pI	UniquepeptideMatches	ProteinCoverage	emPAI
4403	Q53G99	Q53G99_HUMAN	Beta actin variant	635	42080	5.37	11	38.1	1.56
4406	B4DW52	B4DW52_HUMAN	Uncharacterized protein	523	38950	5.19	9	35.2	1.51
4607	P10809	CH60_HUMAN	60 kDa heat shock protein,mitochondrial	453	61187	5.24	9	19.2	0.61
4702	Q53HF2	Q53HF2_HUMAN	Heat shock 70kDa protein 8isoform 2 variant	641	53580	5.62	13	27.4	1.1
4706	E9PNE6	E9PNE6_HUMAN	Uncharacterized protein	185	55170	5.51	4	10	0.3
5407	P06732	KCRM_HUMAN	Creatine kinase M-type	332	43302	6.27	6	22	1.49
5604	Q5SU16	Q5SU16_HUMAN	Beta 5-tubulin	300	50095	4.78	7	16	0.81
5604	Q71U36	TBA1A_HUMAN	Tubulin alpha-1A chain	129	50788	4.94	3	8.2	0.24
6003	P13693	TCTP_HUMAN	Translationally-controlledtumor protein	78	19697	4.84	2	15.7	0.43
6604	Q13885	TBB2A_HUMAN	Tubulin beta-2A chain	667	50274	4.78	12	29.7	1.36
7603	P07237	PDIA1_HUMAN	Protein disulfide-isomerase	362	57480	4.76	7	17.5	0.55
7804	P14625	ENPL_HUMAN	Endoplasmin	1034	92696	4.76	21	27	1.27
8204	P62258	1433E_HUMAN	14-3-3 protein epsilon	492	29326	4.63	9	42.7	1.98
8404	P26641	EF1G_HUMAN	Elongation factor 1-gamma	103	50429	6.25	2	6.2	0.15

Spot number, UniProt protein ID and accession code, protein name, Mascot score, theoretical protein mass and pI values, number of peptide matches, sequence coverage and emPAI values are listed for each protein.

### Validation by Western Blot Analysis

**Figure 3:**
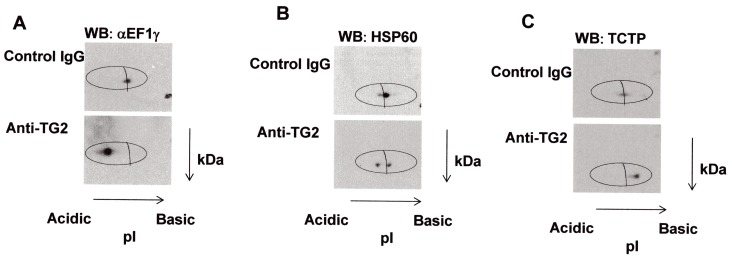
Validation of differential phosphorylation levels by western blot analysis. Total protein extracts (150 µg) from Caco-2 cells treated with anti-TG2 antibodies or with control IgG were separated by 2-DE using linear IPG strips, pH 5.5–6.7 for EF1γ (**A**), pH 4.7–5.9 for HSP60 (**B**), pH 3.9–5.1 for TCTP (**C**). Specific proteins were identified by immunoblot analysis.

To validate proteomic data on differential phosphorylation, we performed western blot analysis on independent protein extracts from Caco-2 cells, treated with anti-TG2 antibodies, or with control IgG, which were previously resolved on 2-D gels by using strips casted with immobilines having narrow pH ranges. For this analysis, we chose TCTP from those proteins that appeared hypophosphorylated (fold change = 0.34), and HSP60 and EF1γ from those proteins that appeared hyperphosphorylated proteins (fold change = 8.36 and 5.75, respectively). After 2-DE and electroblotting, immunostaining with the corresponding primary antibody revealed a pI shift for each protein spot (Figure 3). In particular, we found a shift versus more acidic pH values for EF1γ and HSP60 in samples from anti-TG2-treated cells compared to control samples (Figure 3A and B), thus confirming an increase in protein negative charge (compatible with a higher phosphorylation level) in anti-TG2-treated cells. On the other hand, we observed a shift versus more basic pH values for TCTP in samples from anti-TG2-treated cells compared to control samples (Figure 3C), thus confirming a decrease in protein negative charge (compatible with a lower phosphorylation level) in anti-TG2-treated cells.

### Bioinformatics Analysis of Phosphorylation Sites

The analysis of protein phosphorylation sites for those proteins here identified by MS (excluding the uncharacterized ones) showed that each species has, at least, one potential phosphorylation site (Table S3). For some proteins, the presence of this post-translational modification was documented by several publications, whereas in other cases, only putative sites were predicted. In most cases, there was a consensus between literature data on phosphorylated residues directly identified by MS analysis and those obtained by predictors. In general, proteins reported in this study seemed to act as targets of protein kinase C and, less frequently, of protein kinase A and protein kinase CK2 (for endoplasmin only).

For EF1γ, HSP60 and TCTP, further validated by western blotting, the potential phosphorylation sites were mapped onto the crystallographic structures or available models (Figure S1). Most of these sites are located on flexible loops easily accessible to kinases, and generally they are not involved in functionally characterized parts of the proteins.

### Bioinformatics Analysis of Functional Categories and Pathways of Identified Phosphoproteins

The analysis of the functional categories assigned to the identified proteins allowed us to identify the most common Gene Ontology (GO) classifications in terms of molecular function (MF), biological process (BP) and cellular component (CC) (Table S4). For MF, most proteins were involved in structural activity, or in binding, or they possess catalytic activity; for BP, the most represented processes were metabolic processes, protein folding, immune system processes, developmental processes and cellular component organization. For CC, the proteins were classified as intracellular and forming complexes. In particular, most proteins belonged to the class of chaperone or cytoskeletal proteins, with elements also involved in signalling, nucleotide binding and transferase activity.

In addition, we investigated the physiological pathways in which these proteins are involved (KEGG’s pathways in Table S4). For example, 14-3-3 protein ε and HSP70 are included in the signalling pathway of PI3K-AKT and MAPK, respectively, which are activated by many types of cellular stimuli or toxic insults and are involved in the regulation of fundamental cellular functions, such as transcription, translation, proliferation, growth and survival. In addition, endoplasmin and PDI share a common pathway, i.e. protein processing in endoplasmic reticulum (ER). Finally, the three tubulins identified in this study, as components of the cytoskeleton, are all involved in phagosome and gap junction formation.

We also performed an analysis of the interactors of the proteins identified in this study, and we found that the three hypophosphorylated ones shared two partner proteins, namely the epidermal growth factor receptor (Uniprot code: P00533) and the autophagy protein 5 (Uniprot code: Q9H1Y0). In addition, we found that the two tubulins belonging to the same spot (beta 5-tubulin and tubulin alpha-1A chain, spot 5604 in Table 1) not only share 49 partners, but they also interact each other. The tubulin alpha-1A chain interacts with the tubulin beta-2A chain. Finally, HSP60 and HSP70 protein 8 isoform 2 variant share 7 interactors, which are mainly other HSPs.

## Discussion

Several data support the concept that anti-TG2 antibodies, which are abundant in celiac intestinal mucosa, play an active role in CD pathogenesis [42]–[44]. They may inhibit intestinal epithelial cell differentiation by inhibiting TG2-mediated activation of TGF-β [26]. They also interact with cell-surface TG2, thus promoting cell cycle progression in mucosal CD enterocytes and actin cell cytoskeleton modifications [29], [45]. In addition, anti-TG2 antibodies affect angiogenesis [46], influence handling of gliadin peptides [29], [47] and modulate epithelial barrier function [48]. Notably, an increased cell proliferation, a reduced differentiation, an altered cell and tissue morphology, a compromised barrier function and a less well organized vascular network all represent hallmarks of CD mucosal lesions [13], [49]. Finally, we have provided evidence that anti-TG2 antibodies rapidly mobilize calcium ions from ER and mitochondria in Caco-2 cells. The consequent intracellular calcium increase is sufficient to activate intracellular TG2-transamidating activity and may potentially regulate other calcium-dependent enzymes and signalling pathways [30]. Together, these findings suggest that anti-TG2 antibodies may regulate several intracellular pathways that can collectively contribute to the mucosal damage in CD.

A key mechanism for the regulation of several biological processes, including cell proliferation, differentiation and metabolism, is reversible protein phosphorylation [50]. In this study, we characterized the cellular response to celiac anti-TG2 antibodies by analyzing the differential phosphorylation status of proteins in cells treated or not with specific anti-TG2 antibodies. We performed our experiments on a human intestinal epithelial cell line (Caco-2), which is considered to be a good model for studies on CD. By using a proteomics-based approach that combined phosphoprotein-specific staining and MS analysis, we identified 14 proteins showing significant differences of phosphorylation level in cells treated with anti-TG2 antibodies *vs* cells treated with control unspecific antibodies. 2-D western blot analysis using strips casted with immobilines having narrow pI ranges confirmed changes in proteins pI values, which were consistent with increased phosphorylation for EF1γ and HSP60, and decreased phosphorylation for TCTP in anti-TG2-treated cells. The shift towards more negative charge could be compatible also with the TG2-mediated deamidation. Both EF1γ and HSP60, but not TCTP, have previously been described as acyl-donor TG2 substrates, but there is no evidence that they possess reactive glutamines [51]. Therefore, we are confident that the shift versus more acidic pH values we observed could not be due to a TG2-mediated glutamine deamidation.

Structural analysis of phosphorylation sites for EF1γ, HSP60 and TCTP showed that only a few of them are located in well-structured portions of the protein three-dimensional arrangement; in many cases, instead, the target phosphorylation sites occurred in disordered parts of the protein. Their phosphorylation could therefore induce conformational variations capable of influencing structural and functional properties of the proteins.

The analysis of phosphorylation sites for all proteins identified by MS (excluding uncharacterized proteins) showed that, for many of them, data in the literature support the evidence of phosphorylated forms of human proteins. In three cases, however, there are no direct references in the literature for the presence of human phosphorylated forms, but there is a consensus of predicted phosphorylated sites among different predictors, and this strongly enhances the probability that these residues are indeed phosphorylated in the human protein. The kinases predicted to act on the identified proteins (mainly protein kinase C and A, and CK2 for endoplasmin only) are all part of signal transduction cascades involved in cell growth, proliferation, transformation, apoptosis and senescence, and in mediating immune responses [52], [53]. On the other side, the increase of the intracellular calcium ions level, induced by the anti-TG2-antibodies treatment, let us to suppose that the recently described serine/threonine kinase activity of TG2 could not be responsible of proteins phosphorylation reported in the present work. In fact, it has been demonstrated that calcium inhibits TG2-kinase activity in a dose-dependent manner [8], [54].

Analysis of the functional categories for the set of differentially phosphorylated proteins here identified may contribute to provide some information about the possible metabolic and signalling pathways in which autoimmune responses to TG2 could be involved in CD patients.

Among hyperphosphorylated proteins, we found molecular chaperones (HSP70-isoform 8, HSP60) and proteins with chaperone-like activity (PDI) [55]–[57]. Interestingly, some works have demonstrated a role for phosphorylation in regulating chaperoning functions of these proteins and their ability to interact with client proteins [58], [59]. In addition, a recent study reports that phosphorylation of HSP70-isoform 8 in yeast may regulate cell cycle progression influencing G1 cyclin [60].

Anti-TG2 antibodies also induce an increase of the phosphorylated form of the creatine kinase M-type, a cytosolic enzyme that plays a central role in energy transduction in several tissues [61], and of EF1γ, a subunit of the guanine nucleotide exchange complex EF1Bαγ, which regulates the translational and non-translation activity of the eukaryotic EF1A [62]. Notably, it has been reported that the functions of both these proteins may be regulated by changes in phosphorylation status [63], [64].

In this study, we also found hyperphosphorylated forms of some structural constituents of cytoskeleton, i.e. tubulin alpha-1A, tubulin beta-1B, beta 5-tubulin and beta actin. Phosphorylation of unpolymerized tubulin or actin is thought to perturb their assembly into polymers, thus affecting the overall dynamic properties of cellular microtubules and microfilaments [65], [66]. All of these observations are compatible with the cytoskeleton rearrangement that we observed in Caco-2 cells and in other cell lines after anti-TG2 antibodies treatment [29], [45].

Finally, anti-TG2 antibodies reduce the phosphorylation level of two proteins involved in several cell physiological events, i.e. TCTP and 14-3-3 protein ε and of endoplasmin, an ER-resident member of the HSP90 family, that is implied in the unfolded protein response following ER-stress [67]–[69]. As a cytoskeletal-related protein, TCTP is able to bind to microtubules during cell division, stabilizing the mitotic spindle and thus regulating cell proliferation. In this context, its function is regulated by a phosphorylation/dephosphorylation cycle [70]. Instead, phosphorylation of 14-3-3 protein ε seems to have an important regulatory role, mainly influencing interaction with partner proteins or dimer formation [71]. Interestingly, in the PI3K-AKT pathway,14-3-3 ε inhibits the protein FOXO, which promotes the cell cycle progression. Thus, it is possible that anti-TG2 antibodies exert their pro-proliferative function by modulating the phosphorylation level of both TCTP and 14-3-3 protein ε. On the other hand, the presence of hypophosphorylated endoplasmin may represent a sign of an initial ER-stress response in Caco-2 cells, as already described for GRP78, another abundant ER-resident protein. This is not surprising, since anti-TG2 antibodies rapidly mobilize calcium ions from ER [30] and ER-calcium deprivation is considered a common cause of ER-stress.

In conclusion, the recognition of a differential phosphorylation of several proteins downstream TG2-antibody stimulation supports the concept that these antibodies may act as signaling molecules in intestinal epithelial cells. The functional consequence of differential phosphorylation in CD mucosal environment remains to be established. However, most identified proteins in Caco-2 cells act as key regulators of important cellular processes, such as cell cycle progression, unfolded protein response, cytoskeleton organization, cell stress response and apoptosis. Perturbation of one or more of these processes in the intestinal mucosa of CD-predisposed subjects may be a contributing factor in disease establishment and progression. Interestingly, several differentially phosphorylated proteins identified in this study (HSP70 protein 8, HSP60, EF1γ, beta-tubulin, beta-actin) were previously identified as TG2 substrates in Caco-2 cells, together with other proteins with important chaperon functions (HSP70/HSP90 organizing protein, HSP90, GRP78, chaperonin 3) [51]. Some other cytoskeleton proteins have also been reported as TG2 substrates [2]. Since anti-TG2 antibodies may rapidly increase calcium level in cytosol, thus activating intracellular TG2 [30], it is intriguing to suppose that transamidating activity and differential phosphorylation of the same target proteins may represent a new cellular regulatory mechanism, which may be relevant in the context of the CD mucosa, where TG2-autoantibody levels are high and TG2 expression is consistently increased.

## Supporting Information

Figure S1 **Structures of the three proteins validated by western blot analysis.** (A) Model of the structure of HSP60. The position of ATP in the template is shown in dots. (B) NMR structure (model 1) of TCTP. The putative calcium binding site is in the circle. (C) Model of the N-terminal domain (residues 1–216) of EF1γ. (D) Crystallographic structure of the C-terminal domain (residues 276–437) of EF1γ. Secondary structure elements are represented as springs (helices) and arrows (β-strands). The consensus phosphorylation sites are labelled and shown in stick mode. Consensus phosphorylation sites derived from database information only are shown in orange, consensus phosphorylation sites derived from predictors only are shown in cyan and phosphorylation sites obtained by a consensus of both resources are in red.
(DOCX)

Table S1 **Biological effect and properties of affinity purified normal mouse IgG, used as negative control in this study, of the celiac anti-TG2 clone 2.8 miniantibody, and of the commercial anti-TG2 antibody CUB 7402.** All listed effects and properties have been described in Caco-2 cells, except apoptosis (studied in NIH 3T3 fibroblasts and human mucosal enterocytes). Reference numbers refer to the main text of the manuscript.
(DOCX)

Table S2 **Details of the MS analysis on selected spots.**
(XLSX)

Table S3 **Analysis of phosphorylation sites on proteins identified by MS (excluding uncharacterized proteins).**
(DOCX)

Table S4 **Details of functional categories of 12 identified phosphoproteins.**
(DOCX)
