# Supplementary material for: Celiac Anti-Type 2 Transglutaminase Antibodies Induce Phosphoproteome Modification in Intestinal Epithelial Caco-2 Cells
Source: PLoS One. 2013 Dec 31;8(12):e84403. doi: 10.1371/journal.pone.0084403 (PMC3877280; doi:10.1371/journal.pone.0084403)

**Figure S1 Structures of the three proteins validated by western blot analysis**. (A) Model of the structure of HSP60. The position of ATP in the template is shown in dots. (B) NMR structure (model 1) of TCTP. The putative calcium binding site is in the circle. (C) Model of the N-terminal domain (residues 1-216) of EF1γ. (D) Crystallographic structure of the C-terminal domain (residues 276-437) of EF1γ. Secondary structure elements are represented as springs (helices) and arrows (β-strands). The consensus phosphorylation sites are labelled and shown in stick mode. Consensus phosphorylation sites derived from database information only are shown in orange, consensus phosphorylation sites derived from predictors only are shown in cyan and phosphorylation sites obtained by a consensus of both resources are in red.


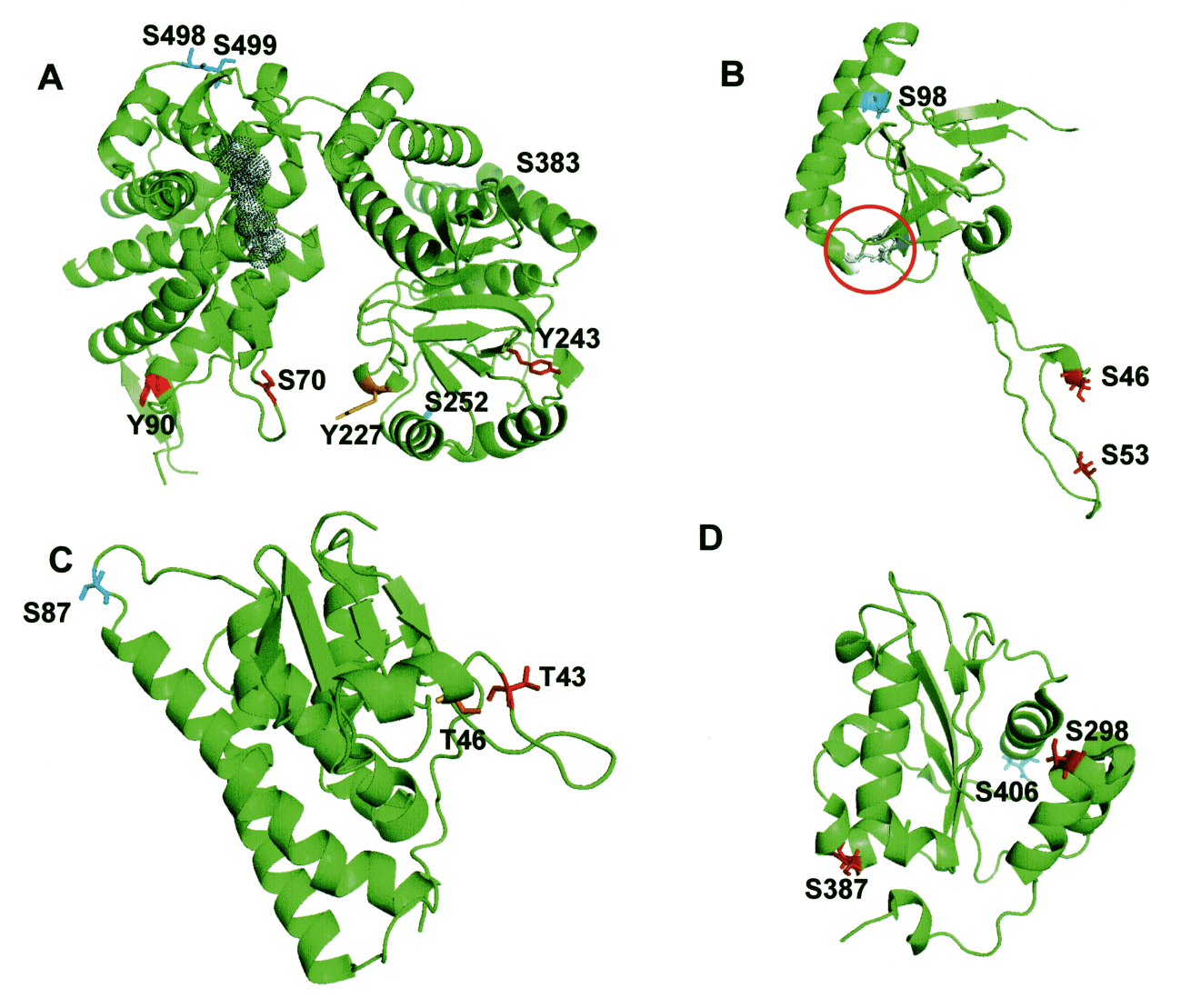

Supplement: Figure S1 — Structures of the three proteins validated by western blot analysis. (A) Model of the structure of HSP60. The position of ATP in the template is shown in dots. (B) NMR structure (model 1) of TCTP. The putative calcium binding site is in the circle. (C) Model of the N-terminal domain (residues 1–216) of EF1γ. (D) Crystallographic structure of the C-terminal domain (residues 276–437) of EF1γ. Secondary structure elements are represented as springs (helices) and arrows (β-strands). The consensus phosphorylation sites are labelled and shown in stick mode. Consensus phosphorylation sites derived from database information only are shown in orange, consensus phosphorylation sites derived from predictors only are shown in cyan and phosphorylation sites obtained by a consensus of both resources are in red. (DOCX) [file pone.0084403.s001.docx]
