# Supplementary material for: Celiac Anti-Type 2 Transglutaminase Antibodies Induce Phosphoproteome Modification in Intestinal Epithelial Caco-2 Cells
Source: PLoS One. 2013 Dec 31;8(12):e84403. doi: 10.1371/journal.pone.0084403 (PMC3877280; doi:10.1371/journal.pone.0084403)
Supplement: Table S1 — Biological effect and properties of affinity purified normal mouse IgG, used as negative control in this study, of the celiac anti-TG2 clone 2.8 miniantibody, and of the commercial anti-TG2 antibody CUB 7402. All listed effects and properties have been described in Caco-2 cells, except apoptosis (studied in NIH 3T3 fibroblasts and human mucosal enterocytes). Reference numbers refer to the main text of the manuscript. (DOCX) [file pone.0084403.s002.docx]

**Table S1. Biological effect and properties of affinity purified normal mouse IgG, used as negative control in this study, of the celiac anti-TG2 clone 2.8 miniantibody, and of the commercial anti-TG2 antibody CUB 7402.** All listed effects and properties have been described in Caco-2 cells, except apoptosis (studied in NIH 3T3 fibroblasts and human mucosal enterocytes). Reference numbers refer to the main text of the manuscript.

| **Biological properties** | **Affinity purified mouse IgG** | **Anti-TG2 clone 2.8** | **Anti-TG2 CUB 7402** |
| --- | --- | --- | --- |
| Induction of actin rearrangement [45] | Not determined | Yes | Yes |
| Activation of intracellular TG2 [30] | No | Yes | Yes |
| Induction of apoptosis [45] | Not determined | No | No |
| Ca^2+^ mobilization from cell deposits [30] | No | Yes | Yes |
| Induction of ERK phosphorylation [29] | No | Yes | Yes |
| Induction of proliferation [29] | No | Yes | Yes |
| Interference with gliadin peptides uptake [29] | No | Yes | Yes |
| Recognition of membrane surface TG2 [29] | No | Yes | Yes |
