# Supplementary material for: Celiac Anti-Type 2 Transglutaminase Antibodies Induce Phosphoproteome Modification in Intestinal Epithelial Caco-2 Cells
Source: PLoS One. 2013 Dec 31;8(12):e84403. doi: 10.1371/journal.pone.0084403 (PMC3877280; doi:10.1371/journal.pone.0084403)
Supplement: Table S3 — Analysis of phosphorylation sites on proteins identified by MS (excluding uncharacterized proteins). (DOCX) [file pone.0084403.s004.docx]

**Table S3.** **Analysis of phosphorylation sites on proteins identified by MS (excluding uncharacterized proteins).**

| **Spot**  **number** | **UniProt Protein Accession codes** | **PDB structure^a^** | **Phosphorylation sites by UniProt^b^** | **Phosphorylation sites by Phosphosite Plus^c^** | **NetPhos 2.0^d^** | **PHOSIDA^e^** | **NetPhosK 1.0^f^** | **Consensus^g^** |
| --- | --- | --- | --- | --- | --- | --- | --- | --- |
| 4403 | Q53G99 (Q53G99_HUMAN) | NO | NO | NO | S52, **Y53,** S**60**, **T**66, S141, T194, S**199**, T202, Y218, S232, S234, **S235, S239**, **Y294,** S323, **Y362** | S52, S60, S145, S155, S232, S233, S234, S235, S239, T304, S348, S350, S365, S368 | NO | **PR**: S52, **S60**, S232, S234, **S235, S239** |
| 4607 | **P10809 (CH60_HUMAN)** | NO (Model: 26-551) | S70, Y227 | **S70**, T79, Y90, **Y227, Y243**, Y385, T409, S410 | S15, **S70, Y90, T164**, T200, **Y223, T231**, Y243, **S247, S252,** T351, **S383**, S498, S499, **Y503** | S15, S70, S83, T117, S122, S132, S159, T163, S252, S253, S256, S383, S398, S410, S453, S488, S497, S498, S499, T547 | n.d. | **DB: S70, Y227** |
|  |  |  |  |  |  |  |  | **PR**: S15, S70, S252, S383, S498, S499 |
|  |  |  |  |  |  |  |  | **BOTH**: S70, Y90, Y243 |
| 4702 | Q53HF2 (Q53HF2_HUMAN) | NO | NO | NO | T38, Y41, S113, T125, **S153**, **T265**, S**275**, S277, **S281, S286, S296**, T**313,** S329, S385, Y431, **T450,** S484, T488 | S113, S120, S121, S221, S254, S275, S276, S277, S281, S286, S329, T341, S362, S385, S400, T418, T429, S432, T462, S480, S484, S485 | S40(PKC), T66(PKC), T145(PKC), S153(PKC), T265(PKG), S275(PKA), S276 (PKC), S340(PKC), T418(PKC) | **PR**: S113, **S153, T265, S275**, S276, S277, **S281**, **S286,** S329, S385, T418, S484 |
| 5407 | P06732 (KCRM_HUMAN) | 1I0E (8-322) | NO | Y14, **Y20**, S24, T35, **Y125,** S128, **Y140, T141**, S164, T166, **Y173, Y174**, S199, **Y279**, T322, S372 | **Y20**, S24, T47, T52, T59, S81, **Y82,** T133, S136, **S158**, **S178**, **S239**, S332, S337, **S344**, **S345**, **S372** | S24, S49, S94, S128, S129, S136, S164, S178, S199, S303, S332, S337, S344, S345, S372 | T6(PKC), T103(PKC), S128(PKC), S129(PKC), T133(PKC), S136(PKC), T322(PKC) | **PR**: S24, S128, S129, T133, S136, **S178**, S332, S337, **S344, S345, S372** |
|  |  |  |  |  |  |  |  | **BOTH**: **Y20, S24,** S128**,** S199, T322, S372 |
| 5604 | Q5SU16 (Q5SU16_HUMAN) | NO | NO | NO | T35, S40, **S48**, S75, Y106, S115, S126, S138, S153, **S172**, T178, Y200, Y222, **T274**, **T285**, T290, **S322**, S382, T409, **S420** | S40, S78, S115, S145, S153, S168, S172, S176, S234, S278, S322, S338, S339, S413 | S382(PKA) | **PR**: S40, S115, S153, **S172, S322** |
| 5604 | Q71U36 (TBA1A_HUMAN) | NO | S48, S439 | **S48, T51, T73, T80, T82, Y103, Y108**, T109, S158**, Y161, Y210, T223, Y224**, T225, S237, **T271, Y272, S277, Y282, S287, Y312, Y319, T334, T337, Y357, T361, Y399, Y432, S439, Y451** | **T82, S158**, **S165**, S170, S198, Y210, **Y224**, S287, S419, **Y432**, **S439**, **Y451** | S147, S151, S165, S170, S198, S236, S237, S241, S419, S439 | T82(PKC), S241(PKC), T337(PKC) | **DB**: **S48,** **S439** |
|  |  |  |  |  |  |  |  | **PR**: **T82**, **S165**, S170, S198, S419, **S439** |
|  |  |  |  |  |  |  |  | **BOTH**: **T82, Y210, Y224,** S237**, T337**, **Y432, S439, Y451** |
| 6003 | **P13693**  **TCTP_HUMAN** | **1YZ1** (1-38 + 67-172), | S46, S53, S64 | **S46**, S53, Y88, Y91, Y95, Y159 | S9, Y18, **S53** | S15, S37, S46, S53, S98 | S98 (PKC) | **DB**: **S46**, S53 |
|  |  | **2HR9** (1-172), |  |  |  |  |  | **PR**: S53, S98 |
|  |  | **3EBM** (1-11 + 13-37 + 67-172) |  |  |  |  |  | **BOTH**: S46, S53 |

| 6604 | Q13885 (TBB2A_HUMAN) | NO | S78, S95 | Y36, **Y50**, S75, S78, **Y106**, T107, S115, **Y183, Y208, T218, T219, T221, Y222**, T274, **S275, S278**, S338, **Y340** | S35, Y59, S75, Y106, S115, **S124**, S126, S138, S153, **S172**, T178, Y200, S201, Y222, **T274**, **T285, S322**, S382, T409, **S420** | S35, S40, S78, S115, S124, S126, S145, S153, S168, S172, S176, S201, S234, S278, S322, S338, S339, S413 | T366(PKC), S382(PKA) | **DB**: S78 |
| --- | --- | --- | --- | --- | --- | --- | --- | --- |
|  |  |  |  |  |  |  |  | **PR**: S35, S115, **S124**, S126, S153, **S172,** S201, **S322**, S382 |
|  |  |  |  |  |  |  |  | **BOTH**: S75, S78, Y106, S115, Y222, T274, S278, S338 |
| 7603 | P07237 (PDIA1_HUMAN) | **1MEK** (18-137), | NO | Y43, Y457 | Y63, **S112**, T139, **S148**, **S152**, **S168**, S194, **S264**, **S266**, **S281**, T319, **T325,** Y327, **S331**, **S357**, Y414, S427, S449, T462 | S32, S75, S88, T110, S112, T138, S148, S152, S153, S166, S168, S188, S190, S194, S264, S266, S293, S331, S427, S472 | S281(PKC) | **PR**: **S112, S152, S168,** S194, **S264, S266, S281, S331** |
|  |  | **1BJX** (136-245), |  |  |  |  |  |  |
|  |  | **2BJX** (136-245), |  |  |  |  |  |  |
|  |  | **3UEM** (137-479), |  |  |  |  |  |  |
|  |  | **3BJ5** (230-368), |  |  |  |  |  |  |
|  |  | **2K18** (135-357), |  |  |  |  |  |  |
|  |  | **1X5C** (368-475) |  |  |  |  |  |  |

| 7804 | P14625 (ENPL_HUMAN) | NO | Y677 | S42, Y94, S106, S109, **S306**, S347, **Y652, Y677, Y678**, T786 | T30, **S38, S42, S73,** Y94, **S119, S127,** T133, **S169**, S172, **S187**, **S213**, **S227**, T240, T246, **Y258**, Y280, **S284**, T288, **S306, T323**, **S347**, **Y355**, **S363**, **S403**, **Y408**, Y429, **Y480**, S515, Y527, **S545**, S551, **S552**, Y567, Y575, **S601**, **S607**, **S637**, T669, Y678, **S680**, S746, **T766**, **T770**, T774, **T792**, **S796** | S18, S38, S42, S64, S73, S106, S109, S119, S127, S169, S172, S187, S189, S225, S227, S231, S256, S283, S284, T288, S306, S347, S363, S366, S403, S406, S439, S501, S514, S515, S544, S545, S551, S552, S601, S607, S643, S725, S746, T770, T774, T786, S796, T797 | S92(PKC), S119(PKA), T165(PKC), T212(PKC), T240(PKA), S306(CKII), **T323(PKC),** T450(PKC), T504(PKC), T669(PKC), S680(PKC), T774(CKII), T786(CKII) | **DB**: **Y677** |
| --- | --- | --- | --- | --- | --- | --- | --- | --- |
|  |  |  |  |  |  |  |  | **PR**: **S38, S42, S73, S119, S127, S169,** S172, **S187, S227,** T240, **S284,** T288, **S306, T323, S347, S363, S403,** S515, **S545,** S551, **S552, S601, S607, S680,** S746, **T770,** T774, T786, **S796** |
|  |  |  |  |  |  |  |  | **BOTH**: **S42,** Y94**,** S106**,** S109**, S119, S306, S347, Y678,** T786 |
| 8204 | P62258 (1433E_HUMAN) | 3UAL (1-232), | T38, S210 | **S46**, Y49, T91, Y122, Y131, T208, **S210**, Y214 | Y20, **T38**, **S59, S64**, Y128, S156, Y182, S187, T208, **S210, S213, Y214** | S64, S65, S156, T161, S210, S213, S217, S233 | S46(PKC), S59(PKA) | **DB**: **S210** |
|  |  | 2BR9 (1-233), |  |  |  |  |  | **PR**: **S64, S210, S213** |
|  |  | 3UBW (1-234) |  |  |  |  |  | **BOTH**: **T38, S46, S210,** T208**, S210, Y214** |
| 8404 | **P26641 (EF1G_HUMAN)** | 1PBU (276-437) (Model: 1-216) | T43, T46 | **T43, T46**, T230, S298, S387 | T43, **S80**, **S87**, **T88**, T230, **S237**, Y297, **S298**, T302, S387, Y394, T398, **S406**, **T411**, **S418** | S33, S87, S104, T230, S237, S298, S304, S324, S368, S369, S370, S372, S406 | NO | **DB**: **T43**, **T46** BOTH: **T43**, T230, **S298**, S387 |
|  |  |  |  |  |  |  |  | **PR**: **S87**, T230, **S237, S298, S406** |
|  |  |  |  |  |  |  |  | **BOTH**: **T43**, T230, **S298**, S387 |

NO: no data. Proteins validated by western blot are shown in bold. ^a^: when the protein structure is available, the PDB code is reported together with the range of sequence covered by structure(s) available. ^b^: only those phosphorylation sites supported by a direct reference for human proteins are taken into account. ^c^: only those phosphorylation sites supported by >1 references for human proteins are shown. Phosphorylation sites supported by >5 references are highlighted in bold. ^d^: Predictions with a threshold >0.7 are shown. Predictions with a threshold >0.9 are in bold. ^e^: Predictions with a threshold >0.95 (default precision for the tool) for only pSer and pThr are shown. ^f^: Phosphorylation sites identified with a threshold >0.7 and positively predicted in a greater subset of the homologues. Predictions with a threshold >0.9 are in bold. Putative kinases are into parentheses for each phosphorylation site. ^g^: Consensus residues are reported separately for data obtained from databases (DB), and for predictors (PR). In the former case, a consensus residue is indicated when both resources are in agreement to indicate the residue as phosphorylated. In the second case, a consensus residue is indicated when at least two out of three predictors agree to indicate the residue as phosphorylated. Consensus residues appearing at least once in both classes (BOTH) are also reported. Data or predictions obtained with higher reliability at least for one resource are highlighted in bold.
