# Supplementary material for: Celiac Anti-Type 2 Transglutaminase Antibodies Induce Phosphoproteome Modification in Intestinal Epithelial Caco-2 Cells
Source: PLoS One. 2013 Dec 31;8(12):e84403. doi: 10.1371/journal.pone.0084403 (PMC3877280; doi:10.1371/journal.pone.0084403)
Supplement: Table S4 — Details of functional categories of 12 identified phosphoproteins. (DOCX) [file pone.0084403.s005.docx]

**Table S4. Details of functional categories of 12 identified phosphoproteins**

| **UniProt Protein Accession codes** | **GO**  **Molecular Function^a^** | **GO**  **Biological Process^a^** | **GO**  **Cellular Component^a^** | **Panther**  **Protein Class** | **KEGG Pathways^b^** |
| --- | --- | --- | --- | --- | --- |
| Q53G99 (Q53G99_HUMAN) | ATP binding* | n.d. | cytoplasm; cytoskeleton* | n.d. | n.d. |
| P10809 (CH60_HUMAN) | chaperone* | protein folding; protein complex assembly | mitochondrion* | chaperonin | hsa03018: RNA degradation |
|  |  |  |  |  | hsa04940: Type I diabetes mellitus |
|  |  |  |  |  | hsa05134: Legionellosis |
|  |  |  |  |  | hsa05152: Tuberculosis |
| Q53HF2 (Q53HF2_HUMAN) | ATP binding* | response to stress* | n.d. | n.d. | hsa03040: Spliceosome |
|  |  |  |  |  | hsa04010: MAPK signaling pathway |
|  |  |  |  |  | hsa04141: Protein processing in ER |
|  |  |  |  |  | hsa04144: Endocytosis |
|  |  |  |  |  | hsa04612: Antigen processing and presentation |
|  |  |  |  |  | hsa04915: Estrogen signaling pathway |
|  |  |  |  |  | hsa05134: Legionellosis |
|  |  |  |  |  | hsa05145: Toxoplasmosis |
|  |  |  |  |  | hsa05162: Measles |
|  |  |  |  |  | hsa05164: Influenza A |
|  |  |  |  |  | hsa05169: Epstein-Barr virus infection |
| P06732 (KCRM_HUMAN) | amino acid kinase activity | muscle contraction; metabolic process | cytoplasm* | amino acid kinase | hsa00330: Arginine and proline metabolism |
|  |  |  |  |  | hsa01100: metabolic pathways |
| Q5SU16 (Q5SU16_HUMAN) | GTP binding; GTPase activity; structural constituent of cytoskeleton* | protein polymerization; spindle assembly | cytoplasm, cytoskeleton, microtubule* | n.d. | hsa04145: Phagosome |
|  |  |  |  |  | hsa04540: Gap junction |
|  |  |  |  |  | hsa05130: Pathogenic Escherichia coli infection |
| Q71U36 (TBA1A_HUMAN) | structural constituent of cytoskeleton | intracellular protein transport; mitosis; cell motion; chromosome segregation; cellular component morphogenesis | tubulin complex | tubulin | hsa04145: Phagosome |
|  |  |  |  |  | hsa04540: Gap junction |
|  |  |  |  |  | hsa05130: Pathogenic Escherichia coli infection |
| P13693 (TCTP_HUMAN) | structural constituent of cytoskeleton; microtubule binding | immune system process | microtubule | non-motor microtubule binding protein | n.d. |
| Q13885 (TBB2A_HUMAN) | structural constituent of cytoskeleton | intracellular protein transport; mitosis; cell motion; chromosome segregation; cellular component morphogenesis | tubulin complex | tubulin | hsa04145: Phagosome |
|  |  |  |  |  | hsa04540: Gap junction |
|  |  |  |  |  | hsa05130: Pathogenic Escherichia coli infection |
| P07237 (PDIA1_HUMAN) | protein disulfide isomerase activity | protein modification process | cell membrane; ER* | isomerase | hsa04141: Protein processing in ER |
| P14625 (ENPL_HUMAN) | chaperone* | immune system process; protein folding; response to stress | ER* | HSP90 family chaperone | hsa04141: Protein processing in ER |
|  |  |  |  |  | hsa04151: PI3K-Akt signaling pathway |
|  |  |  |  |  | hsa04621: NOD-like receptor signaling pathway |
|  |  |  |  |  | hsa04915: Estrogen signaling pathway |
|  |  |  |  |  | hsa05200: Pathways in cancer |
|  |  |  |  |  | hsa05215: Prostate cancer |
| P62258 (1433E_HUMAN) | n.d. | cell cycle; signal transduction | cytoplasm* | chaperone | hsa04110: Cell cycle |
|  |  |  |  |  | hsa04114: Oocyte meiosis |
|  |  |  |  |  | hsa04151: PI3K-Akt signaling pathway |
|  |  |  |  |  | hsa04390: Hippo signaling pathway |
|  |  |  |  |  | hsa04722: Neurotrophin signaling pathway |
|  |  |  |  |  | hsa05169: Epstein-Barr virus infection |
|  |  |  |  |  | hsa05203: Viral carcinogenesis |
| P26641 (EF1G_HUMAN) | oxidoreductase activity; transferase activity; racemase and epimerase activity; structural constituent of cytoskeleton; nucleic acid binding; receptor binding; translation elongation factor activity | immune system process; intracellular signaling cascade; oxygen and reactive oxygen species metabolic process; translation; response to toxin | cytoskeleton | transferase; signaling molecule; reductase; translation elongation factor; epimerase/racemase; cytoskeletal protein | hsa05134: Legionellosis |

n.d.: data not found; ^a^: data with asterisks were obtained from UniProt, whereas data without asterisks were obtained by PANTHER analysis; ^b^: The pathways are described by the KEGG code and definition.
